# Risk Factors for *Clostridium difficile* Infection in Inpatients: A Four-Year (2017–2020) Retrospective Study

**DOI:** 10.3390/antibiotics14020133

**Published:** 2025-01-29

**Authors:** Chu-Hsuan Hsia, Hsiu-Yueh Su, Yi-Wen Chien

**Affiliations:** 1 School of Nutrition and Health Sciences, Taipei Medical University, Taipei 11031, Taiwan; da07108003@tmu.edu.tw (C.-H.H.); hysu@h.tmu.edu.tw (H.-Y.S.); 2 Department of Dietetics, Taipei Medical University Hospital, Taipei 11031, Taiwan; 3 Nutrition Research Center, Taipei Medical University Hospital, Taipei 11031, Taiwan; 4 TMU Research Center for Digestive Medicine, Taipei Medical University, Taipei 11031, Taiwan; 5 Graduate Institute of Metabolism and Obesity Sciences, Taipei Medical University, Taipei 11031, Taiwan; 6 Research Center of Geriatric Nutrition, College of Nutrition, Taipei Medical University, Taipei 11031, Taiwan

**Keywords:** *Clostridium difficile* infection, nutrition status, nutrition route, antibiotic

## Abstract

Background: *Clostridium difficile* infection (CDI) impact on healthcare systems is concerning due to high treatment cost and increased hospitalization time. We found that the incidence rate of CDI increased annually at Taipei Medical University Hospital (TMUH). The study aimed to establish monitoring indicators for hospitalized patients receiving antibiotics to prevent CDI occurrences. Methods: A case–control study was conducted to identify the risk factors of CDI among patients who were admitted to TMUH and tested for *C. difficile*. Patient demographics, patient history, and laboratory data were collected and analyzed. Results: Nutrition Risk Screening 2002 score (NRS 2002) in CDI patients was significantly lower than in non-CDI patients (3.1 ± 1.7 vs. 3.5 ± 1.6). The percentage of tube feeding in CDI patients was significantly lower than in non-CDI patients (23.0% vs. 36.7%), whereas parenteral nutrition was higher (8.8% vs. 3.8%). Age (OR = 1.03, *p* = 0.00), NRS 2002 score (OR =0.86, *p* = 0.05), comorbidity with cardiovascular disease (OR = 1.73, *p* = 0.03) or pulmonary disease (OR = 3.72, *p* = 0.00), patients with tube feeding (OR = 2.02, *p* = 0.01), and the number of medications (OR = 1.15, *p* < 0.01) were significant predictors of CDI. Conclusion: This study revealed that nutritional factors, including NRS 2002 scores and feeding routes, were associated with CDI, emphasizing the importance of nutritional factors as key predictors in managing and preventing CDI.

## 1. Introduction

*Clostridium difficile* infection (CDI) is a leading cause of healthcare-associated infections, characterized by significant morbidity, prolonged hospital stays, and increased healthcare costs worldwide. A study from the United States revealed the number of *C. difficile* infections at 453,000 and deaths at 29,300 in 2011 [1]. In Europe, the incidence of CDI exceeds 120,000 cases per year, and CDI is a major cause of healthcare-associated infection [2,3]. In a medical center in Southern Taiwan, the incidence was 42.6 cases per 100,000 patient days and was highest in intensive care units (110.6 cases per 100,000 patient days) [4]. Nationwide surveillance showed that central Taiwan has the highest incidence of CDI, with an incidence rate of 5.8–7.3 per 10,000 patient days, followed by 4.6–6.3 per 10,000 patient days in northern Taiwan and 1.1–4.8 per 10,000 patient days in southern Taiwan [5]. The overall in-hospital mortality is 20–26.2% [6,7], and the disease-related, in-hospital mortality of community-onset CDI is 4.7% [8]. Also, a review concluded that screening for asymptomatic carriers at hospital admission can theoretically reduce CDI by isolating carriers to reduce transmission and implementing antibiotic stewardship measures targeting carriers to prevent progression to clinical illness [9].

The identified risk factors of CDI were antibiotic exposure, advanced age and hospitalization. Antibiotic exposure increases the risk of CDI; certain classes such as third-/fourth-generation cephalosporins, carbapenems, fluoroquinolones, clindamycin and β-lactamase inhibitor combination penicillin antibiotics have been found to be high-risk [10,11,12]. A case–control study showed that compared to a 7-day course, a 14-day course was associated with a 27% higher risk of CDI. However, a shorter 5-day course was associated with a 9% lower risk [13], suggesting the duration of antibiotic exposure also has a significant impact on CDI. Advanced age is strongly associated with an increased risk of CDI, as elderly patients often have weakened immune systems, multiple comorbidities, and frequent hospitalizations with high exposure to antibiotics, which further contribute to their vulnerability to CDI. A study conducted in Canada found that individuals aged ≥65 years had a 10-fold higher risk of CDI compared to those aged < 65 years [14]. Similarly, a study in Korea concluded that the incidence of CDI has risen significantly among the elderly with comorbidities, with longer hospital stays and higher total medical costs observed in the CDI group [15]. 

A study utilizing Taiwan’s National Intensive Care Unit Database revealed that mechanical ventilation and carbapenem use significantly increase the risk of CDI in critically ill patients, these two factors synergistically contributing to its development [16]. Despite these well-established risk factors, there is growing interest in understanding the role of nutritional status and interventions in influencing CDI risk. For instance, a case–control study conducted in a French university hospital found that malnutrition affected approximately half of the patients with CDI and was associated with a high risk of CDI, likely due to immunodeficiency caused by malnutrition [17]. Additionally, a prospective study using multivariate conditional logistic regression analysis identified older age, recent antibiotic and glucocorticoid treatments, and total parenteral nutrition (TPN) risk factors for CDI [18]. 

However, these studies lacked a standardized definition of malnutrition and did not explore why TPN might increase the risk of CDI. The Nutrition Risk Screening (NRS) 2002 tool is designed to identify undernutrition and the risk of developing undernutrition in hospitalized patients, as it assesses nutritional risk through a composite score that considers nutritional status, disease severity, and age, with a total score ranging from 0 to 7 [19,20]. To provide a clear and standardized assessment of malnutrition, the NRS 2002 offers a potential framework for investigating the link between nutritional factors and CDI risk. Nevertheless, studies exploring nutritional factors, including nutritional status and interventions in CDI risk factors, remain limited. To date, no research has utilized the NRS 2002 as a variable to examine CDI risk factors, highlighting a critical gap in the literature.

We observed the incidence rate of CDI among hospitalized patients increased nearly 3.5 times, from 0.04% in 2017 to 0.14% in 2019 at Taipei Medical University Hospital (TMUH), Taiwan. This study aimed to develop monitoring indicators, with a particular emphasis on nutritional factors, for hospitalized patients receiving antibiotics to help prevent CDI occurrences.

## 2. Results

### 2.1. General Characteristics

During the study period, 2616 stool samples were tested for CDI and 200 samples were confirmed to have CDI. After excluding 12 repetitive samples from 11 patients, a total of 188 unique CDI cases were identified, showing a total incidence rate of 7.1%. Among these patients, 54.8% were male and 63.3% were elderly. A total of 376 non-CDI patients were matched as controls. The mean age of CDI and non-CDI were significant differences between the groups (69.1 ± 17.2 years and 51.5 ± 38.6 years, *p* = 0.02). The proportion of ICU admissions was 44.7% in non-CDI groups, whereas 35.1% in CDI groups. The mean days of diarrhea was 2.5 ± 1.8 vs. 2.0 ± 2.0 days in CDI and non-CDI groups. The mean age, admission to the ICU and the days of diarrhea were significantly different between the groups. The comorbidities such as CVD and pulmonary disease were significant differences between the groups. According to the medication record, we found that the mean duration of the anti-diarrhea drug, fourth-generation cephalosporin, and vancomycin used was significantly longer in CDI patients compared to non-CDI patients. Meanwhile, the number of medications used was 6.5 ± 2.8 in CDI patients and 5.6 ± 3.0 in non-CDI patients, respectively (*p* < 0.001). The demographic and clinical characteristics of these patients are shown in Table 1.

### 2.2. Nutrition-Related Parameters and Nutrition Intake

The NRS 2002 score in CDI patients was 3.1 ± 1.7, which was significantly lower than in non-CDI patients, who scored 3.5 ± 1.6 (*p* = 0.02). Oral intake was observed to have no differences in CDI patients compared to non-CDI patients (48.0% vs. 39.4%). The percentage of tube feeding in CDI patients was significantly lower than in non-CDI patients (23.0% vs. 36.7%, *p* = 0.01), whereas PN was higher (8.8% vs. 3.8%, *p* = 0.03). Additionally, CDI patients received EN plus PN, which was significantly higher than non-CDI patients (2.7% vs. 0.3%, *p* = 0.04). There were no significant differences in laboratory data between CDI and non-CDI patients (Table 2). Regarding the nutritional intake of our study groups, the actual calorie intake of the 1st visit was 1044.4 ± 502.5 kcal (57.4 ± 34.5%) in CDI patients and 1121.9 ± 465.1 kcal (54.6 ± 37.6%) in non-CDI patients. These values significantly increased to 1340.3 ± 419.7 kcal (83.0 ± 24.8%) and 1420.9 ± 353.8 kcal (81.5 ± 28.5%), respectively at the second visit. Similar results were observed in actual protein intake, with significant increases between the first and second visits in both groups. However, there was only a significant difference in actual caloric intake at the second visit between CDI and non-CDI patients (1340.3 ± 419.7 kcal vs. 1420.9 ± 353.8 kcal, *p* =0.03) but not in the percentage of the actual intake (87.6% vs. 85.3%, *p* =0.35) (Table 3).

### 2.3. Predictors of CDI

A binary logistic regression was conducted to assess the predictors of CDI. The results of the univariate and multivariate logistic regression analyses are presented in Table 4. In univariate analysis, age (OR = 1.02, *p* = 0.00), NRS 2002 score (OR = 0.88, *p* = 0.05), ICU admission (OR = 1.49, *p* = 0.03), hospital stay (OR = 1.01, *p* = 0.05), and number of medications (OR = 1.11, *p* < 0.01) were significant predictors of CDI. Comorbidity with cardiovascular disease (OR = 1.69, *p* = 0.01) or pulmonary disease (OR = 3.76, *p* = 0.00), as well as patients with tube feeding (OR = 1.78, *p* = 0.01) or using PN (OR = 0.41, *p* = 0.03), were also significant predictors of CDI. We also conducted two models of multivariate analyses to predict CDI. In Model 1, ICU admission, the use of PN, and hospital stay were not significant predictors. In Model 2, only ICU admission was not a significant predictor. Age (OR = 1.03, *p* = 0.00), NRS 2002 score (OR = 0.86, *p* = 0.05), comorbidity with cardiovascular disease (OR = 1.73, *p* = 0.03) or pulmonary disease (OR = 3.72, *p* = 0.00), patients with tube feeding (OR = 2.02, *p* = 0.01), and number of medications (OR = 1.15, *p* < 0.01) remained significant predictors.

## 3. Discussion

Our study examined nutritional status as a potential risk factor for developing CDI, a topic that has been less discussed in previous studies. We found that CDI patients were much older, had longer durations of diarrhea, and were on more medications compared to non-CDI patients, which is consistent with previous studies [11]. Patients over 65 years old have a 5- to 10-fold increased risk for CDI compared to patients under 65 years old. Moreover, age over 65 was not only a significant risk factor for CDI itself but also in poor clinical outcomes, including severity and mortality. Ampicillin, amoxicillin, cephalosporin, clindamycin, and fluoroquinolones are the antibiotics that were most frequently associated with CDI [11,21,22], as our study has found that the number of antibiotics used was higher. The duration of 4th-generation cephalosporin and vancomycin use was significantly longer in CDI patients than in non-CDI patients. According to the recommendations and guidelines of CDI treatment in Taiwan, prescribing 125 mg of vancomycin four times per day for 10 days was recommended, which further explains the longer duration of vancomycin use in CDI groups. We also found the proportion of ICU admission, CVD and pulmonary disease was significantly higher in non-CDI patients than in CDI patients. Patients with CVD or pulmonary disease are more likely to be admitted to ICU because these conditions increase the risk of deterioration or lead to severe complications, as a previous retrospective study in Taiwan has found that the categories leading to ICU admission include cardiovascular systems (21.6%) and respiratory systems (17.6%) [23].

Supportive care, with attention to correcting fluid losses and electrolyte imbalances, is important in treating CDI as illness-associated ranges from mild diarrhea to life-threatening colitis and toxic megacolon. In severe cases, patients are initially placed on complete bowel rest, and nasogastric tube decompression may be required at the discretion of the treating clinician; furthermore, PN may be necessary for patients who cannot tolerate enteral nutrition [24,25]. We found that the number of PN and EN + PN patients was significantly higher in CDI than in non-CDI patients, which indicates that diarrhea was more severe in CDI patients, as evidenced by their longer durations of diarrhea. Enteral feeding can influence gut physiology by altering intestinal transit time, modifying secretory and absorptive functions, and disrupting microbial ecology [26]. These changes may disturb the gut microbiota, reducing colonization resistance and potentially contributing to the development of diarrhea, which could increase the risk of CDI [27].

Additionally, the presence of food in the gut lumen is a vital stimulus for mucosal cell growth. In contrast, parenteral nutrition (PN) bypasses the gastrointestinal tract and may lead to gut atrophy and reduced microbiome diversity, increasing gut permeability and potentially associating with CDI [28,29]. According to the European Society for Clinical Nutrition and Metabolism (ESPEN) guidelines, calorie delivery should be gradually increased to 80–100% of the measured energy expenditure within 3 days in the ICU [30]. In both CDI and non-CDI patients, we found that their actual calorie and protein intake met 50–60% of the requirement at the 1st visit and reached 80% of the requirement at the 2nd visit as assessed by a dietitian, regardless of ICU admission. Although there were no differences in the percentage of calorie and protein intake between both groups of patients, significant differences in actual calorie intake were observed. This suggests that nutritional assessment in CDI patients should focus on actual calorie intake rather than the percentage of macronutrients.

Consistent with previous studies, factors such as advanced age, ICU admission, longer hospital stays, and polypharmacy were identified as significant predictors of CDI in the univariate analysis [11,12,22,31]. Furthermore, a notable distinction of our study is the inclusion of nutritional factors, specifically the assessment of nutritional risk (NRS 2002 score) and the route of nutritional support (tube feeding or PN). A systematic review and meta-analysis article demonstrated a statistically significant association between feeding tube insertion and risk of severity or complicated CDI, with a 1.81-fold increased risk of developing severity or complicated CDI [32]. We found the inverse association between NRS 2002 scores and CDI risk, indicating that patients with lower nutritional risk (NRS 2002 score < 3) had a higher likelihood of CDI compared to those with higher nutritional risk (NRS 2002 score ≥ 3). Possible explanations are as follows. (1) Patients identified as being at high nutritional risk are often targeted for intensive nutritional support and medical interventions, such as supplemental feeding or closer monitoring. These proactive measures may help mitigate the risk of CDI through improved overall health and gut microbiome stability. (2) The NRS 2002 scoring system combines disease severity and nutritional status. In some cases, patients with lower scores may still have underlying risks that are not fully captured by the scoring system. (3) Our retrospective study may have exhibited information bias and selection bias. Additionally, as CVD and pulmonary disease were significant predictors of CDI, it is possible that their management indirectly influenced nutritional risk scoring. Previous studies have shown that HF patients with CDI are associated with longer lengths of hospital stay compared to those without CDI and have significantly higher in-hospital mortality rates [33]. Another study conducted in the United States found that among patients admitted with ST-elevation myocardial infarction (STEMI) complicated by cardiogenic shock, the most common nosocomial infections were urinary tract infections, hospital-acquired pneumonia, central line-associated bloodstream infections, bacteremia, skin-related infections, and CDI [34]. Although antibiotic therapy is an essential part of management for patients with aspiration pneumonia, a retrospective cohort study has found that antibiotic therapy with extended anaerobic coverage provided no additional mortality benefit in aspiration pneumonia but was associated with an increased risk of CDI [35]. 

Broad-spectrum antibiotics such as amoxicillin with clavulanic acid or a macrolide are commonly used as first-line treatment in the treatment of acute exacerbations of COPD [36], which are recognized risk factors for CDI. A meta-analysis including 118 studies demonstrated that half of the infections in acute exacerbations of COPD patients are bacterial [37] and might require active antibiotic treatment [38]. Taken together, these findings might explain why CVD and pulmonary disease emerged as significant predictors in the present study, which supports and highlights the need for targeted preventive strategies in these high-risk populations. A systematic review concluded a significantly increased risk of poor clinical outcomes of CDI in patients with CKD but not in those with end-stage renal disease (ESRD) [39]. Nevertheless, in our study, neither CKD nor ESRD was identified as a significant predictor of CDI. Potential explanations for these differences include variations in the definitions of CKD and ESRD, differences in confounder adjustment methods, and the fact that all included studies were observational in nature. Hence, after adjusting for confounders in the multivariate analysis, only age, NRS 2002 score, comorbidities with CVD or pulmonary disease, tube feeding and polypharmacy remained significant predictors, suggesting these factors independently contribute to CDI risk. These findings highlight the importance of considering nutritional factors in CDI risk, including nutrition status and feeding methods, which have not been extensively explored in prior CDI research, thereby providing new insights into the role of nutritional intervention in managing and potentially preventing CDI.

Several limitations were found in our study; first, this is a single-center, retrospective case–control study, which may be subject to information and selection bias. As a result, the findings may not be generalizable to other populations or healthcare settings. Also, differences in patient demographics, institutional protocols for CDI prevention, and antibiotic stewardship programs could affect the generalizability of our results. Further multi-center studies with diverse patient populations and standardized protocols are needed to validate these findings across different healthcare environments. Second, while nutritional variables were included, we lacked detailed data on patients’ diet or specific nutritional needs, which may have affected the interpretation of the results. Third, the relatively small sample size of CDI cases limits the statistical power of the analysis, particularly for complex interactions among variables. Additionally, while we included previously identified confounders, such as age, comorbidities, hospitalized days and medication use, in the multivariable regression model, other potentially relevant factors—such as specific formula or environmental exposures—were not assessed, which may influence CDI risk and warrant further exploration. Fourth, the unexpected finding that lower NRS 2002 scores were associated with higher CDI risk warrants further investigation to clarify the underlying mechanisms. Lastly, our study did not assess potential temporal changes in CDI risk factors or healthcare practices over the study period. Future research should address these limitations through prospective, multi-center studies with larger sample sizes and more comprehensive data collection.

In this study, we identified advanced age, ICU admission, comorbidities with CVD or pulmonary disease, longer hospital stays, and polypharmacy as significant predictors of CDI. In particular, the findings underscore the critical role of nutritional factors, such as NRS 2002 scores and the route of nutritional support, in influencing CDI risk. The hospital infection control team should integrate nutrition risk monitoring and feeding route evaluation into CDI prevention strategies. Screening with NRS 2002 can identify high-risk patients early, such as optimizing nutritional support and minimizing unnecessary tube feeding. For dietitians, the emphasis should be on personalized nutrition plans to maintain gut integrity and microbiome stability, reducing CDI risk. These findings underscore the need for a holistic approach addressing both clinical and nutritional factors in CDI management and prevention.

## 4. Materials and Methods

### 4.1. Study Design and Definition

We performed a case–control study to identify the risk factors of CDI among patients admitted to TMUH. The study subjects were adult patients admitted between 1 January 2017 and 31 December 2020 who underwent testing for *C. difficile*. Exclusion criteria were age less than 18 years old, hospital stays less than two days, outpatients, patients who did not reside in long-term care facilities within the past six weeks and patients who were diagnosed with CDI within 72 h of admission [40]. CDI was defined as the presence of gastrointestinal symptoms such as diarrhea, abdominal distension, or abdominal pain in patients receiving antibiotics, with positive toxin A/B in stool samples. We used the propensity score matching (PSM) method to ensure a robust comparison. Two controls were randomly selected from hospitalized patients with a negative *C. difficile* toxin test, matched by gender, age group (18–40, 41–65, and > 65 years old), and the year of the stool test. The Ethics Review Board of Taipei Medical University approved this study on 30 September 2020 (approval number N202009013).

### 4.2. Study Variables and Data Collection

We collected and analyzed patient demographics, patient history, medication data, and laboratory information through a retrospective chart review, ensuring data quality by employing trained personnel. Demographic variables included height, weight, age, gender, positive *C. difficile* toxin sample date, and length of hospital stay. Patient history included comorbidities such as diabetes mellitus (DM), chronic kidney disease (CKD), dialysis, cancer, cardiovascular disease (CVD), pulmonary disease, and inflammatory bowel disease, including ICU admission history and frequency of diarrhea. CVD cases included conditions such as heart failure (HF), coronary artery disease, and myocardial infarction, while pulmonary disease cases included conditions such as aspiration pneumonia, bronchopneumonia, chronic obstructive pulmonary disease (COPD), coronavirus disease 2019 (COVID-19) and empyema. The medication record included types and dosages of antibiotics, proton pump inhibitors, and histamine-2 blockers. Nutritional-related variables were assessed using the NRS 2002 score and the route of nutritional support, such as oral intake, enteral nutrition (EN), or parenteral nutrition (PN). Calorie and protein intake during hospitalization were recorded by a registered dietitian. Laboratory data, collected from 4 days before to 2 days after the first *C. difficile* test sample was submitted, included nutritional-related biochemical values such as hemoglobin, white blood cells (WBC), neutrophils, C-reactive protein (CRP), albumin, and electrolytes. Other variables included the length of hospital stay and clinical or imaging findings recorded during the hospitalization period before the diagnosis of CDI, such as colitis, pericolic stranding, and abnormal gastrointestinal symptoms.

### 4.3. Statistical Analysis

The variables were presented as mean ± standard deviation (SD), numbers or percentages. The normality of continuous variables was assessed using the Shapiro–Wilk tests. For comparisons between CDI and non-CDI groups, continuous variables were analyzed using the Student’s *t*-test if they met the normal distribution criteria and the Mann–Whitney U test if they did not. Categorical variables were examined using Pearson’s Chi-squared test or Fisher’s exact test. Univariable and multivariable logistic regression models were used to obtain the odds ratios (ORs) and corresponding 95% confidence intervals (CIs) for predicting. To select appropriate predictors or variables for the multivariable logistic regression models, all significant predictors (*p* < 0.05) from the univariable analysis were included in the multivariable logistic regression as Model 1. The model was then adjusted by applying backward elimination, resulting in Model 2. For all tests performed, two-tailed *p*-values < 0.05 were considered statistically significant. SPSS version 26.0 was used for the statistical analysis.

## Figures and Tables

**Table 1:** Basic characteristics of CDI and non-CDI patients.

	All	CDI (*n* = 188)	Non-CDI (*n* = 376)	*p*-Value
**Gender**				
Male	309	103 (54.8%)	206 (54.8%)	1.00
Female	255	85 (45.2%)	170 (45.2%)	1.00
**Age**	57.4 ± 34.0	69.1 ± 17.2	51.5 ± 38.6	0.02 *
18–40 yr	39	13 (6.9%)	26 (6.9%)	1.00
41–65 yr	161	56 (29.8%)	105 (27.9%)	0.64
> 65 yr	364	119 (63.3%)	245 (65.2%)	0.66
**Body mass index (BMI) (kg/m^2^)**	22.9 ± 4.7	22.6 ± 4.8	23.1 ± 4.6	0.21
Underweight	88	31 (16.5%)	57 (15.2%)	0.64
Normal weight	259	94 (50.0%)	168 (44.7%)	0.23
Overweight	119	35 (18.6%)	84 (22.3%)	0.31
Obesity	95	28 (14.9%)	67 (17.8%)	0.38
**ICU admission**	234 (41.5%)	66 (35.1%)	168 (44.7%)	0.03 *
**Days of diarrhea**	2.1 ± 1.9	2.5 ± 1.8	2.1 ± 2.0	0.03 *
**Hospitalization days**	29.3 ± 27.8	32.5 ± 32.5	27.7 ± 25.0	0.22
**Comorbidity**				
Cancer	180	68 (36.2%)	112 (29.8%)	0.13
Cardiovascular disease	227	61 (32.4%)	166 (44.1%)	0.01 *
Diabetes mellitus	167	51 (27.1%)	116 (30.9%)	0.29
Pulmonary disease	231	41 (21.8%)	190 (51.2%)	0.00 *
Inflammatory bowel disease	6	2 (1.1%)	4 (1.1%)	1.00
Renal disease_CKD	76	24 (12.8%)	52 (13.8%)	0.73
Renal disease_Dialysis	48	14 (7.4%)	34 (9.0%)	0.39
**Medication record (days)**				
H2-blocker	10.3 ± 15.9	10.4 ± 18.4	10.2 ± 14.6	0.26
Proton pump inhibitor	13.5 ± 22.8	15.7 ± 26.9	12.4 ± 20.4	0.33
Anti-diarrhea	9.0 ± 13.4	12.0 ± 17.4	7.5 ± 10.6	0.01 *
Antibiotics				
Broad-spectrum penicillin	7.2 ± 12.4	6.8 ± 15.3	7.4 ± 10.7	0.12
Clindamycin	0.5 ± 2.3	0.5 ± 2.5	0.5 ± 2.2	0.92
1st-generation cephalosporin	1.0 ± 3.4	0.9 ± 3.2	1.1 ± 3.5	0.60
2nd-generation cephalosporin	1.7 ± 3.9	1.6 ± 3.4	1.8 ± 4.1	0.83
3rd-generation cephalosporin	4.7 ± 9.1	4.8 ± 7.7	4.6 ± 9.7	0.49
4th-generation cephalosporin	1.6 ± 4.3	2.2 ± 5.0	1.3 ± 3.8	0.05 *
Macrolide	0.8 ± 6.4	0.5 ± 1.9	0.9 ± 7.7	0.87
Penicillinase-sensitivity penicillin	1.0 ± 4.8	1.0 ± 4.5	1.0 ± 5.0	0.79
Aminoglycoside	2.6 ± 6.6	2.8 ± 6.5	2.5 ± 6.6	0.94
Vancomycin	5.0 ± 12.6	10.3 ± 18.7	2.4 ± 6.7	0.00 *
**Medication using days**	23.8 ± 26.2	26.1 ± 30.7	22.6 ± 23.7	0.73
**Numbers of medication**	5.9 ± 2.9	6.5 ± 2.8	5.6 ± 3.0	0.00 *

ICU—intensive care unit; CKD—chronic kidney disease. * *p* < 0.05 showed significant differences between groups by using the Mann–Whitney U test (continuous variables) and Chi-square test (categorical variables).

**Table 2:** Nutrition-related parameters of CDI and non-CDI patients.

	All	CDI	Non-CDI	*p*-Value
**NRS 2002 score**	3.4 ± 1.6	3.1 ± 1.7	3.5 ± 1.6	0.02 *
**Nutrition risk**				
Well-nourished	7	4 (2.7%)	3 (1.0%)	0.23
Mild malnutrition risk	109	41 (27.5%)	68 (23.4%)	0.29
Moderate malnutrition risk	246	79 (53.0%)	167 (57.4%)	0.59
Severe malnutrition risk	78	25 (16.8%)	53 (18.2%)	0.80
**Nutrition route**				
Nothing by mouth (NPO)	76	26 (17.6%)	50 (17.3%)	0.86
Enteral nutrition (EN)				
Oral intake	185	71 (48.0%)	144 (39.4%)	0.08
Tube feeding	140	34 (23.0%)	106 (36.7%)	0.01 *
Parenteral nutrition (PN)	24	13 (8.8%)	11 (3.8%)	0.03 *
EN + PN	5	4 (2.7%)	1 (0.3%)	0.04 *
**Laboratory data**				
WBC (10^3^/uL)	10.1 ± 6.3	10.1 ± 6.5	10.1 ± 6.2	0.79
Hemoglobin (g/dL)	10.6 ± 2.7	10.5 ± 2.3	10.6 ± 2.3	0.60
% of Neutrophil	76.5 ± 13.9	75.7 ± 14.9	76.9 ± 13.4	0.56
Albumin (g/dL)	3.0 ± 0.7	3.0 ± 0.7	3.1 ± 0.4	0.41
CRP (mg/dL)	8.5 ± 9.1	8.1 ± 7.9	8.6 ± 9.6	0.65
Na (mEq/L)	136.5 ± 8.6	135.5 ± 11.6	137.0 ± 6.6	0.17
K (mEq/L)	3.8 ± 0.8	3.8 ± 0.8	3.8 ± 0.8	0.62

NRS 2002—nutrition risk screening 2002; EN—enteral nutrition; PN—parenteral nutrition; WBC—white blood cells; CRP—C-reactive protein. * *p* < 0.05 showed a significant difference between groups by using the Mann–Whitney U test.

**Table 3:** Nutrition intake of CDI and non-CDI patients.

	CDI	Non-CDI	*p*-Value
Actual calorie intake_1st visit (kcal)	1044.4 ± 502.5	1121.9 ± 465.1	0.24
Actual protein intake_1st visit (g)	43.8 ± 21.9	47.3 ± 20.3	0.14
Actual calorie intake_2nd visit (kcal)	1340.3 ± 419.7	1420.9 ± 353.8	0.03 *
Actual protein intake_2nd visit (g)	59.8 ± 24.4	63.0 ± 16.9	0.16

Data analyzed by using the Mann–Whitney U test. * *p* < 0.05 showed a significant difference.

**Table 4:** Univariate and multivariate analysis of factors associated with CDI.

	Univariate Analysis		Multivariate Analysis (Model 1) ^a^		Multivariate Analysis (Model 2) ^b^	
Characteristics	OR (95% CI)	*p*-Value	OR (95% CI)	*p*-Value	OR (95% CI)	*p*-Value
**Gender**	1.00 (0.70–1.42)	1.00				
**Age**	1.02 (1.01–1.02)	0.00 *	1.03 (1.02–1.04)	0.00 *	1.03 (1.02–1.04)	0.00 *
**BMI**	0.98 (0.94–1.02)	0.22				
**NRS 2002 score**	0.88 (0.78–1.00)	0.05 *	0.86 (0.74–1.00)	0.05 *	0.86 (0.74–0.99)	0.05 *
**Admit to ICU**	1.49 (1.04–2.14)	0.03 *	1.56 (0.96–2.55)	0.07	1.57 (0.97–2.56)	0.07
**Days of diarrhea**	1.13 (0.99–1.28)	0.07				
**Comorbidity**						
Cancer	0.75 (0.52–1.09)	0.13				
Cardiovascular disease	1.69 (1.17–2.44)	0.01 *	1.72 (1.05–2.81)	0.03 *	1.73 (1.06–2.81)	0.03 *
Diabetes mellitus	1.23 (0.83–1.82)	0.29				
Pulmonary disease	3.76 (2.52–5.62)	0.00 *	3.67 (2.25–5.99)	0.00 *	3.72 (2.28–6.06)	0.00 *
Inflammatory bowel disease	1.00 (0.18–5.51)	1.00				
Renal disease_CKD	1.09 (0.65–1.84)	0.73				
Renal disease_Dialysis	1.34 (0.69–2.60)	0.39				
**Nutrition route**						
NPO	0.96 (0.57–1.59)	0.86				
Oral intake	0.72 (0.50–1.04)	0.08				
Tube feeding	1.78 (1.15–2.75)	0.01 *	1.96 (1.16–3.31)	0.01 *	2.02 (1.20–3.41)	0.01 *
PN	0.41 (0.18–0.92)	0.03 *	0.53 (0.20–1.40)	0.20		
EN + PN	0.12 (0.01–1.10)	0.06				
**Length of hospital stay**	1.01 (1.00–1.01)	0.05 *	1.00 (0.99–1.01)	0.47		
**Stool status (form X days)**	1.02 (0.99–1.05)	0.12				
**Number of medications**	1.11 (1.05–1.18)	<0.001 *	1.12 (1.01–1.24)	0.04 *	1.15 (1.06–1.25)	<0.001 *
**Medication using days**	1.01 (0.99–1.01)	0.13				

BMI—body mass index; NRS 2002—nutrition risk screening 2002; ICU—intensive care unit; CKD—chronic kidney disease; NPO—nothing by mouth; PN—parenteral nutrition; EN—enteral nutrition. ^a^ Model 1 included all significant predictors from the multivariable analysis. ^b^ Model 2 selected variables through backward elimination. Stepwise and forward selection processes selected the same variables. * *p* < 0.05 showed a significant difference.

## Data Availability

The raw data supporting the conclusions of this article will be made available by the authors upon request.

## Abbreviations

The following abbreviations are used in this manuscript:

CDI	*Clostridium difficile* infection
TPN	Total parenteral nutrition
NRS 2002	Nutrition Risk Screening 2002
DM	Diabetes mellitus
CKD	Chronic kidney disease
CVD	Cardiovascular disease
HF	Heart failure
COPD	Chronic pulmonary disease
NPO	Nothing by mouth
CEN	Enteral nutrition
PN	Parenteral nutrition
BMI	Body mass index
ESRD	End-stage renal disease
COVID-19	Coronavirus disease 2019
